# A Comparative Study on Two Territorial Fishes: The Influence of Physical Enrichment on Aggressive Behavior

**DOI:** 10.3390/ani11071868

**Published:** 2021-06-23

**Authors:** Zonghang Zhang, Yiqiu Fu, Zhen Zhang, Xiumei Zhang, Shengcan Chen

**Affiliations:** 1 The Key Laboratory of Mariculture, Ministry of Education, Ocean University of China, Qingdao 266003, China; zonghangzhang@126.com (Z.Z.); 17860826125@163.com (Y.F.); ouczhangz@163.com (Z.Z.); 2 Fisheries College, Zhejiang Ocean University, Zhoushan 316022, China; 3 Laboratory for Marine Fisheries Science and Food Production Processes, Qingdao National Laboratory for Marine Science and Technology, Qingdao 266237, China; 4 National Fisheries Technology Extension Center, Beijing 100125, China; ziyuanyhc@126.com

**Keywords:** *Sebastes schlegelii*, *Hexagrammos otakii*, environmental enrichment, aggressive behavior, fish welfare

## Abstract

**Simple Summary:**

This study aimed to evaluate the effect of physical enrichment levels (i.e., the intensity of physical enrichment) on the aggressive behavior of two territorial fishes, black rockfish (*Sebastes schlegelii*) and fat greenling (*Hexagrammos otakii*). The main results show that with the increase in the enrichment level, the frequency of aggressive behavior of black rockfish gradually decreased. In contrast, a non-monotonous effect of the enrichment level on aggression was observed for fat greenling, with low and intermediate levels leading to no or more aggression, while a high enrichment level reduced aggression. After three days, the high-level enrichment groups in both rockfish and greenling reached social stability (i.e., a relatively stable social structure indicated by low aggression), while aggression in the other groups continued to increase. These results verify the regulatory effect of enrichment levels on the aggressive behavior in both black rockfish and fat greenling. This study may provide useful information for reducing fish aggression and improving fish welfare in aquaculture.

**Abstract:**

Intraspecific aggression is detrimental to body/fin damage, physiological stress, and other problems in aquaculture. Environmental enrichment has been proposed to have positive effects on fish aggressive behavior, physiological stress, and fish welfare, but there are mixed results. Here, we examine the impact of physical enrichment levels (i.e., the intensity of physical enrichment) on aggression in black rockfish (*Sebastes schlegelii*) and fat greenling (*Hexagrammos otakii*). Generally, with the increase in the enrichment level, the frequency of the aggressive behavior of black rockfish gradually decreased. In contrast, a non-monotonous effect of the enrichment level on aggression was observed for fat greenling, with low and intermediate levels leading to no or more aggression, while a high enrichment level reduced aggression. After three days, the high-level enrichment groups in both rockfish and greenling reached social stability (i.e., a relatively stable social structure indicated by lower aggression), while aggression in the other groups continued increased. These results show the significant regulatory effect of enrichment levels on the aggressive behavior in both black rockfish and fat greenling. This study may promote the development of environmental enrichment measures, and it provides useful information for reducing fish aggression and improving fish welfare in aquaculture.

## 1. Introduction

In nature, intraspecific competition is an important driving force for species/individual distribution, adaptive evolution, and community stability [1,2,3,4]. In aquaculture, however, intraspecific competition (and aggressive behavior in particular) is often seen as a negative factor that should be avoided [5,6,7]. A large amount of studies have showed that intraspecific aggression often results in severe body/fin damage, increased physiological stress, decreased feeding appetite, more food waste, reduced growth, lower immune function and disease resistance, and even cannibalism and mortality [8,9,10,11,12]. In recent years, fish welfare has gained increasing attention from the scientific community and the public [6,7,13,14,15]. Although there is no full consensus on what constitutes fish welfare, it is agreed that aggressive behavior typically lowers the welfare of fish [6]. In fact, preventing aggressive behavior among fish is a top priority for the improvement of fish welfare in aquaculture.

In recent years, environmental enrichment has been proposed to have huge potential for reducing aggressive behavior and physiological stress and improving the welfare of captive fish [9,16]. Environmental enrichment can be categorized into several types, for example, physical enrichment, social enrichment, sensory enrichment, and occupational enrichment [9,17]. Among these, physical enrichment is the most commonly used type [9,17]. Physical enrichment can be simply defined as introducing objects (e.g., physical structures, plants, and substrates) into the housing environment with the aim of increasing environmental complexity [18]. In fact, a series of studies were conducted to explore the enrichment effect on the aggressive behavior of fish, but the results were considerably mixed, i.e., positive [8,10,19,20,21,22], negative [23,24,25], and no effects [26,27,28]. These discrepancies may be related to species-specific effects, developmental stage, enrichment mode (type, level, and color), and methodological differences. Recently, we verified the regulatory effects of enrichment levels (i.e., the intensity of physical enrichment) on the behavior and physiology in the territorial fish *Sebastes schlegelii* [11,29,30], but whether such effect exists in other fish species remains unclear. It is important to examine whether or not the intensity of physical enrichment determines fish aggression in order to develop effective animal welfare measures.

The black rockfish (*Sebastes schlegelii*) and fat greenling (*Hexagrammos otakii*) are two typical territorial fish species and are widely distributed in the Northwestern Pacific, especially in the coastal waters of China, Korea, and Japan [10,31]. In recent years, aquaculture of these two fishes in northern China has rapidly developed, primarily as a source of food and for stock enhancement [30,32]. However, during the rearing process, severe aggressive behavior and subsequent physiological stress and even high mortality are often observed, and identifying a way to efficiently reduce aggression is now of upmost urgency. Traditional rearing environments in aquaculture do not include any physical structures as shelters, which may not only harm fish welfare considering the fact that these fish have the natural instinct of territoriality in the wild, but also strip the opportunity for subordinates to hide from aggression [11]. In this study, we aimed to explore whether the enrichment level is an effective modulator for fish aggression and to compare the potential difference in the behavioral response to physical enrichment between black rockfish and fat greenling.

## 2. Materials and Methods

Before conducting this study, we read the policies relating to animal experiments and animal welfare and confirmed that this study complied with them (ARRIVE guidelines; EU Directive 2010/63/EU for animal experiments). All procedures performed in this study were approved by the Institutional Animal Care and Use Committee of the Ocean University of China (identification code 20180101).

### 2.1. Animals and Experimental Design

**Figure 1:**
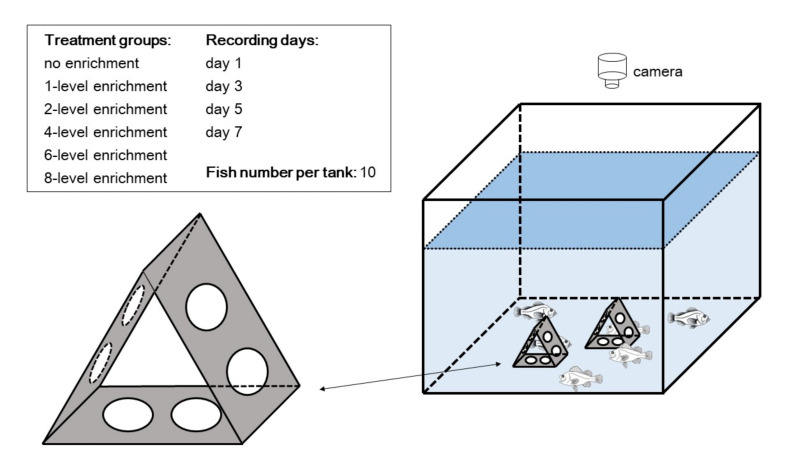
Sketches of overall experimental design and physical structure used for environmental enrichment. The treatment groups include different enrichment levels (i.e., intensity of enrichment; no, 1-level, 2-level, 4-level, 6-level and 8-level enrichments). This figure shows the 2-level enrichment treatment. The number of physical structures depends on the experimental treatment.

Black rockfish (*S. schlegelii)* juveniles (mean body length, 11.24 ± 0.07 cm; mean body weight, 22.82 ± 0.30 g) and fat greenling (*H. otakii*) juveniles (mean body length, 11.56 ± 0.07 cm; mean body weight, 17.23 ± 0.34 g) were obtained from a commercial hatchery (Weihai City, Shandong Province, China) and acclimated to the experimental conditions for one week before the formal experiments. This study included two similar experiments, and apart from the fish species (black rockfish vs. fat greenling), all experimental conditions were identical. In brief, 180 fish per species were randomly divided into 18 glass tanks (60 cm × 50 cm × 50 cm) in groups of 10, in 6 triplicated treatments. We created six experimental groups by introducing different amounts of physical structures into each tank [10,11,29]. Considering that each tank included 10 fish, introducing eight physical structures was deemed to be sufficient for fish to occupy the structures (to express their natural instinct) as well as to support free swimming in the tank [11]. Therefore, in this study, we designed the six treatments as no structure per tank, one structure per tank, two structures per tank, four structures per tank, six structures per tank, and eight structures per tank (hereafter, we simply refer to the treatments as no enrichment, 1-level enrichment, 2-level enrichment, 4-level enrichment, 6-level enrichment, and 8-level enrichment, respectively). We selected this pyramidal physical structure (color: grey, shape: pyramid, height: approximately 10 cm, basal area: approximately 72 cm^2^; Figure 1) as the enrichment object mainly for the following reasons: (i) the shape and color are compatible with the natural habitat of black rockfish and fat greenling, (ii) the material does not chemically interact with rearing water, and (iii) this structure was shown to have positive effects on the behavior and physiology of black rockfish [10,11,29].

Fish were maintained under experimental conditions for seven days. The glass tanks were part of an indoor flow-through seawater system equipped with mechanical filters, and each tank was provided with an air stone to ensure a high dissolved oxygen content (6.9 ± 0.1 mg/L, 98.5 ± 0.3% saturation). The water flow rate was 2 L/min, and the water depth was maintained at 40 cm. The photoperiod followed the natural day–night cycle (12 h light/12 h dark), and the water temperature was maintained at 18.1 ± 0.2 °C. The fish were fed commercial floating dry pellets (moisture, ≤10.0%; crude protein, ≥50.0%; crude lipid, ≥9.0%; crude ash, ≤16.0%; crude fiber, ≤2.0%; total phosphorus, 1.5–3.0%; lysine, ≥2.5%) to satiation by hand once daily (09:00 a.m.). The feces and residual fodders were siphoned once daily before feeding. The adjacent sides of the tanks were masked with white plastic boards to ensure visual separation. The tanks were placed on gray mats. No fish died during the experimental period.

### 2.2. Behavioral Observations

Based on our personal experience and careful observations, these two territorial fishes are relatively inactive compared to other fish species, and our previous work showed there was no significant difference in locomotor activity among barren and enriched groups [11]. Therefore, we mainly focused on fish aggressive behavior, which may be the most typical behavior in territorial fishes. During the seven-day rearing period, aggressive behavior was recorded at days 1, 3, 5, and 7 for each tank using a digital camera (HDR-AS100V, Sony Corporation, Tokyo, Japan; Figure 1). For each day, a 10 min video was filmed between 11:00 a.m. and 14:00 p.m. from the top side of each tank, and the distance between the tank and camera was approximately 50 cm. Per tank, aggressive behavior was quantified by counting the total amount of chasing, nipping, and biting among fish, and the identification of specific behaviors was based on our previous descriptions [11,33,34]. The collection and analysis of behavioral data were conducted by one person who was blind to the experimental design (i.e., Zhen Zhang).

### 2.3. Data Analysis

Based on our experimental design per fish species, we obtained a series of data from six treatments (i.e., no enrichment, 1-level enrichment, 2-level enrichment, 4-level enrichment, 6-level enrichment, and 8-level enrichment) at four time points (i.e., days 1, 3, 5, and 7). We were primarily interested in the changing trends within different treatments and within different time points. Because the number of aggressive encounters is count data, we performed generalized linear models with Poisson error distribution. Treatment, recording day, and their interaction were added as fixed factors to examine the differences among treatments at each recording day and among recording days within each treatment. The differences were detected by Tukey-corrected post-hoc multiple comparisons. All statistical analyses were conducted using R software for Microsoft Windows. The R codes were uploaded as Supplementary Material. Differences were considered significant at *p* < 0.05. All values in the text and figures are expressed as means ± S.E.

## 3. Results

### 3.1. Enrichment Effect on the Aggressive Behavior in Black Rockfish

**Figure 2:**
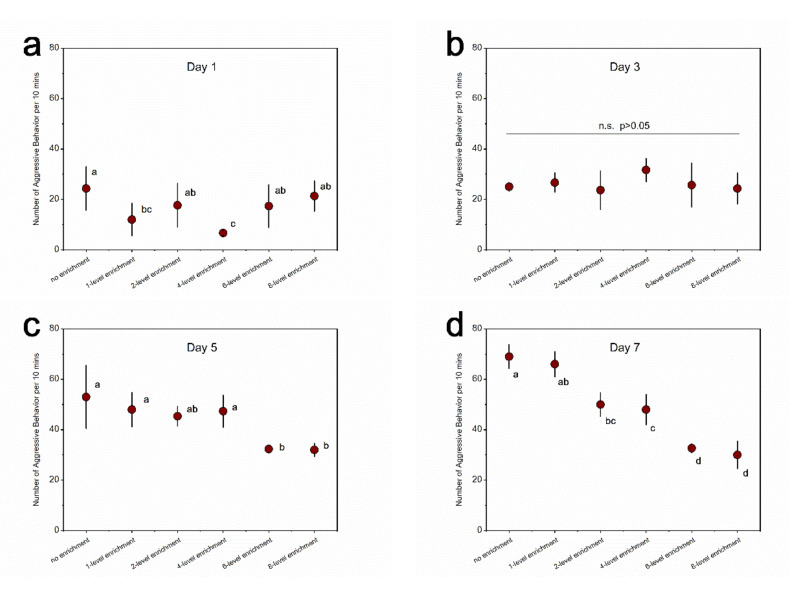
Levels of aggressive behavior in black rockfish among treatments within specific experimental days. (**a**) Day 1; (**b**) day 3; (**c**) day 5; (**d**) day 7. Different letters indicate significant differences among treatments within a specific experimental day. n.s. indicates no significant difference. *n* = 3. Data are presented as means ± S.E.

For the aggressive behavior in black rockfish, the significant main and interactive effects of treatment and recording day were detected (treatment: χ^2^ = 69.02, df = 5, *p* < 0.0001; day: χ^2^ = 382.16, df = 3, *p* < 0.0001; interaction: χ^2^ = 86.10, df = 15, *p* < 0.0001). There was no significant difference among treatments on day 3 (Figure 2; Supplementary Material). However, on day 5, the aggressive behavior of the 6-level and 8-level enrichment groups was significantly lower than that of the no, 1-level, and 4-level enrichment groups (Figure 2; Table S1). On day 7, the no and 1-level enrichment groups presented the most aggressive behavior, while the 6-level and 8-level enrichment groups were the least aggressive among the six groups (Figure 2; Table S1). No significant differences were detected between the 6-level and 8-level enrichment groups (Figure 2; Table S1).

**Figure 3:**
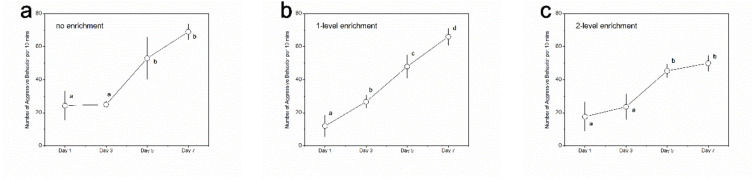

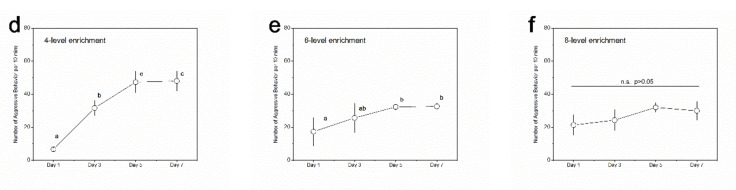
Levels of aggressive behavior in black rockfish among experimental days within a specific treatment group. (**a**) No enrichment group; (**b**) 1-level enrichment group; (**c**) 2-level enrichment group; (**d**) 4-level enrichment group; (**e**) 6-level enrichment group; (**f**) 8-level enrichment group. Different letters indicate significant differences among experimental days within a specific treatment group. n.s. indicates no significant difference. *n* = 3. Data are presented as means ± S.E.

In terms of the changing trends of aggressive behavior among different recording days, the 1-level enrichment group showed significantly higher aggression on day 7 than that on day 5 (Figure 3; Table S2). No significant differences were detected among the latter two recording points (i.e., day 5 and day 7) in the no, 2-level and 4-level enrichment groups; among the latter three recording points (i.e., day 3, day 5, and day 7) in the 6-level enrichment group; and among all four recording points in the 8-level enrichment group (Figure 3; Table S2). These results indicate that the no, 2-level, and 4-level enrichment groups reached social stability after five days of interactions; the 6-level enrichment group reached social stability after three days of interactions; and the 8-level enrichment group reached social stability after one day of interactions, but the 1-level enrichment group did not reach stability even after five days of interactions.

### 3.2. Enrichment Effect on the Aggressive Behavior in Fat Greenling

**Figure 4:**
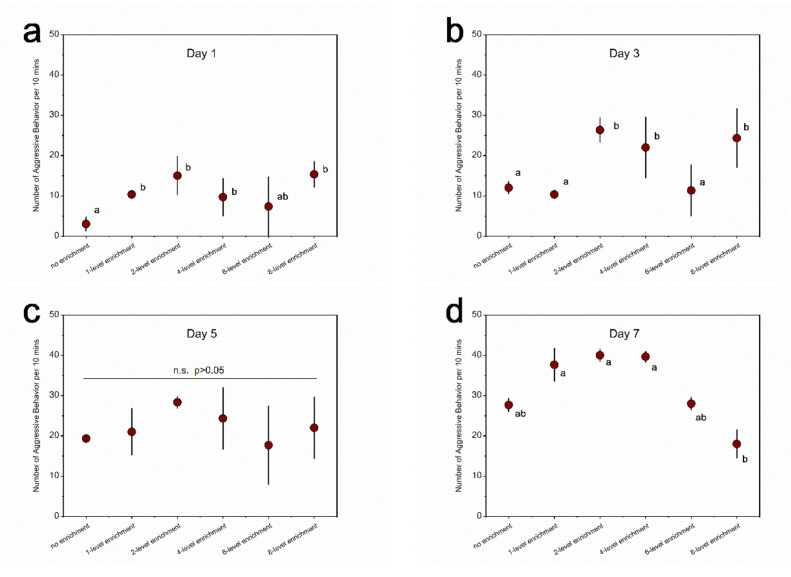
Levels of aggressive behavior in fat greenling among treatments within a specific experimental day. (**a**) Day 1; (**b**) day 3; (**c**) day 5; (**d**) day 7. Different letters indicate significant differences among treatments within a specific experimental day. n.s. indicates no significant difference. *n* = 3. Data are presented as means ± S.E.

For the aggressive behavior in fat greenling, the significant main and interactive effects of treatment and recording day were detected (treatment: χ^2^ = 60.568, df = 5, *p* < 0.0001; day: χ^2^ = 222.385, df = 3, *p* < 0.0001; interaction: χ^2^ = 69.842, df = 15, *p* < 0.0001). There was no significant difference among treatments on day 5 (Figure 4; Table S3). However, on day 7, the 1-level, 2-level, and 4-level enrichment groups had the highest level of aggressive behavior, the no and 6-level enrichment groups presented moderate aggressive behavior, and the 8-level enrichment group was the least aggressive among the six groups (Figure 4; Table S3). 

**Figure 5:**
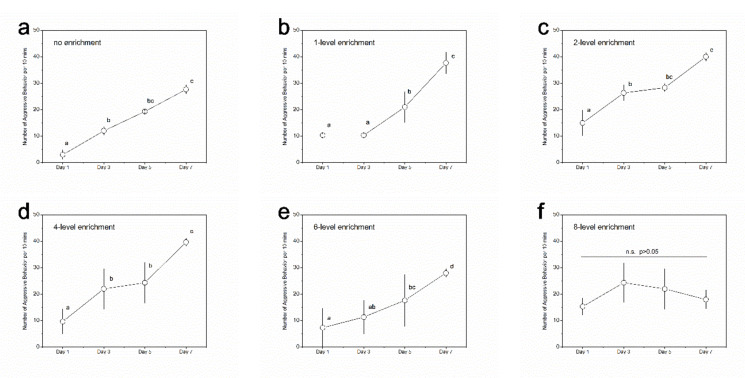
Level of aggressive behavior in fat greenling among experimental days within a specific treatment group. (**a**) No enrichment group; (**b**) 1-level enrichment group; (**c**) 2-level enrichment group; (**d**) 4-level enrichment group; (**e**) 6-level enrichment group; (**f**) 8-level enrichment group. Different letters indicate significant differences among experimental days within a specific treatment group. n.s. indicates no significant difference. *n* = 3. Data are presented as means ± S.E.

In terms of the changing trends of aggressive behavior in different recording days, the 1-level, 4-level, and 6-level enrichment groups showed significantly higher aggression on day 7 than that on day 5 (Figure 5; Table S4). No significant difference was detected among the latter two recording points in the no and 2-level enrichment groups or among all four recording points in the 8-level enrichment group (Figure 5; Table S4). These results indicate that the 8-level enrichment group reached social stability after one day of interacting, but other enrichment groups did not.

## 4. Discussion

The main results of this study are as follows: (i) with the increase in the enrichment level, the aggressive behavior of black rockfish gradually decreased; (ii) for fat greenling, the 1-level, 2-level, and 4-level enrichment groups presented the highest level of aggressive behavior, the no and 6-level enrichment groups were moderately aggressive, and the 8-level enrichment group displayed the lowest level of aggressive behavior among the six groups; (iii) the 6-level and 8-level enrichment groups in rockfish had reached social stability after socially interacting for three days, while the aggressive behavior in the other groups continued to increase; (iv) similarly, after interacting for one day, the 8-level enrichment group in greenling reached social stability, while the aggressive behavior in the other groups continued to increase. These results indicate that the enrichment level had major effects on the aggressive behavior in rockfish and greenling, but the enrichment effects between the two territorial fishes had some differences. Next, we discuss a potential regulatory effect of the enrichment level on fish aggression from a comparative perspective.

The effects of physical enrichment on fish aggressive behavior have been explored in several studies, but there are considerably mixed results [9,16]. For example, in Atlantic salmon (*Salmo salar*), barren-reared fish had higher levels of fin deterioration than those of fish from physically enriched environments, which is potentially due to higher aggression levels [35]. Through direct behavioral observations, it was shown that physical enrichment could significantly decrease aggressive behavior in redbreast tilapia (*Tilapia rendalli*) [21] and seabream (*Sparus aurata*) [8]. In turquoise killifish (*Nothobranchius furzeri*), physical enrichment during the rearing of fish affected boldness and exploration but had no effect on fish aggressiveness later in life [36]. In contrast, others found that structurally complex environments significantly increased aggressive behavior in Eurasian perch (*Perca fluviatilis*) [37] and zebrafish (*Danio rerio*) [23]. Our results clearly show that the enrichment level is an important moderator of effect direction and magnitude. Firstly, physical structures were introduced into the rearing environment as shelter [9]. These shelters divided the whole water into several relatively separate spaces, obstructed the visual contact among fish, decreased fish encounter frequency, restricted territorial size, increased territory number, and consequently reduced intraspecific aggression [19,20,38,39]. This effect could be called the “shelter effect”. Secondly, territorial fish have the natural instinct of maintaining a specific territory and competing for the resources within it [30]. Naturally, the physical structures were a valuable resource of occupancy, so the physical enrichment sometimes stimulated fish aggression [25,40]. This effect could be called the “resource effect”. Thirdly, the introduction of structures inevitably caused some fish to occupy the shelters, while others inhabited the “open area”. It was observed that the number of fish in the open area was less than the whole fish stock, and this may have increased familiarity among the fish in the open area. Previous studies showed that familiarity often decreases the aggressive behavior of fish and improves their growth [18,41,42]. Therefore, physical enrichment may also decrease fish aggression through this “familiarity effect”.

Based on the above three potential mechanisms, we could reasonably interpret the enrichment results on fish aggression. For greenling, the introduction of one, two, or four physical structures may not have provided sufficient shelter, and in this situation, the resource effect was stronger than both the shelter and familiarity effects. The fish competed for resources and finally showed increased aggressive behavior [11]. In contrast, introducing eight physical structures may be sufficient for most fish to use as shelter, thus weakening the resource effect, and, finally, the 8-level enrichment group displayed the lowest level of aggression [9]. However, for rockfish, we did not observe such a phenomenon (i.e., an initial increase followed by a decrease in aggression) but rather a gradually decreasing trend. This may be because the rockfish at this developmental stage had a relatively milder temperament than that of greenling; that is, the greenling had stronger territoriality than that of rockfish. Therefore, introducing six physical structures into the environment of the rockfish resulted in the lowest intraspecific aggression. This result is in accordance with our previous study, in which we showed that the low–medium-level structural enrichment could significantly reduce aggressive behavior and physiological stress in black rockfish [11]. Interestingly, in the same study, we used plastic plants as enrichment objects for black rockfish and observed a similar trend both in aggressive behavior and stress physiology to that of greenling in the current study, i.e., an initial increase followed by a decrease in aggression [11]. This further verified that the abovementioned three mechanisms may also exist in rockfish, but the outcomes may be related to enrichment type (i.e., enrichment levels and enrichment types have interactive effects).

Another interesting finding was that compared with no enrichment group, proper enrichment (e.g., the 6-level and 8-level enrichment groups in rockfish, and the 8-level enrichment group in greenling) could significantly accelerate the formation of social stability, while specific enrichment (e.g., the 1-level enrichment group) may slow down this process. This result indicates that the time duration to reach social stability is related to enrichment intensity, and the direction and magnitude may depend on trade-offs among resource, shelter, and familiarity effects. One previous study showed that for zebrafish (*Danio rerio*), aggression remained high during days 1–5 in tanks containing glass structures before falling to a lower level by day 7, but in barren tanks, this lower level was reached 2 days earlier [43]. The authors concluded that the glass structures may have slowed down the rate of establishment of a social structure. We speculate that the longer time duration to establish stability in this study may result from the resource effect being stronger than both the shelter and familiarity effects. Moreover, we speculate that the greenling had stronger territoriality than that of rockfish, considering the fact that the 6-level enrichment group in rockfish had stabilized by day 3, while this group in greenling did not. Notably, in the present study, we concentrated on the aggressive behavior of fish mainly because it may be the most typical behavior of our target fish, but this does not mean that aggression is the only modulated behavior by enrichment. A large amount of studies have shown that physical enrichment affects not only aggression but also other behaviors that are relevant to fish welfare, such as locomotor activity, exploration, boldness, and anti-predator behavior [9,16,18]. The welfare parameters measured by future research should depend on their research aims and the behavioral habits of their target animals. 

## 5. Conclusions

In conclusion, our study shows that the effect of physical enrichment is intensity dependent, and the latency time to reach social stability is dependent on the intensity of enrichment. Moreover, our results suggest that the territoriality of greenling is stronger than that of rockfish. We strongly suggest that future research and practices fully consider the fish species and enrichment level when they intend to conduct environmental enrichment. Further research should explore the interactive effects of enrichment levels and other factors and should verify the enrichment effects when using more fish species from a comparative perspective. Our study may promote the development of environmental enrichment measures, and it provides useful information for reducing fish aggression and improving fish welfare in aquaculture.

## Data Availability

All of the data and analyzed codes have been uploaded as Supplementary materials.

## Supplementary Materials

The following are available online at https://www.mdpi.com/article/10.3390/ani11071868/s1, Table S1: The statistical results of the number of aggressive behavior in black rockfish among treatments within specific recording day; Table S2: The statistical results of the number of aggressive behavior in black rockfish among recording days within specific treatment group; Table S3: The statistical results of the number of aggressive behavior in fat greenling among treatments within specific recording day; Table S4: The statistical results of the number of aggressive behavior in fat greenling among recording days within specific treatment group; Supplementary Data: All raw data of this study, Supplementary Codes: The analyzed R codes used for this study.
