# Supplementary material for: A Comparative Study on Two Territorial Fishes: The Influence of Physical Enrichment on Aggressive Behavior
Source: Animals (Basel). 2021 Jun 23;11(7):1868. doi: 10.3390/ani11071868 (PMC8300207; doi:10.3390/ani11071868)
Supplement: Supplementary file 1 [file animals-11-01868-s001.zip › Supplementary Material.pdf]

A comparative study on two territorial fishes: the influence of physical enrichment on aggressive behavior

Zonghang Zhang [1], Yiqiu Fu [1], Zhen Zhang [1], Xiumei Zhang [2,3,*], Shengcan Chen [4]

[1] The Key Laboratory of Mariculture, Ministry of Education, Ocean University of China, Qingdao 266003, China; zonghangzhang@126.com (Z.Z.); 17860826125@163.com (Y.F.); ouczhangz@163.com (Z.Z.)

[2] Fisheries College, Zhejiang Ocean University, Zhoushan 316022, China; xmzhang1227@163.com (X.Z.)

[3] Laboratory for Marine Fisheries Science and Food Production Processes, Qingdao National Laboratory for Marine Science and Technology, Qingdao 266237, China

[4] National Fisheries Technology Extension Center, Beijing 100125, China; ziyuanyhc@126.com (S.C.)

[*] Correspondence: xmzhang1227@163.com

Table S1.

The statistical results of the number of aggressive behavior in black rockfish among treatments within specific recording day.

Record day	Contrast	Estimate	z.ratio	p.value
Day 1	(0-level) vs. (1-level)	0.7069	3.471	0.0069
	(0-level) vs. (2-level)	0.3202	1.774	0.4826
	(0-level) vs. (4-level)	1.2947	5.130	<.0001
	(0-level) vs. (6-level)	0.3392	1.869	0.4212
	(0-level) vs. (8-level)	0.1316	0.768	0.9728
	(1-level) vs. (2-level)	-0.3868	-1.791	0.4717
	(1-level) vs. (4-level)	0.5878	2.108	0.2832
	(1-level) vs. (6-level)	-0.3677	-1.696	0.5344
	(1-level) vs. (8-level)	-0.5754	-2.762	0.0638
	(2-level) vs. (4-level)	0.9746	3.714	0.0028
	(2-level) vs. (6-level)	0.0190	0.098	1.0000
	(2-level) vs. (8-level)	-0.1886	-1.015	0.9130
	(4-level) vs. (6-level)	0.9555	-3.632	0.0038
	(4-level) vs. (8-level)	-1.1632	-4.540	0.0001
	(6-level) vs. (8-level)	-0.2076	-1.112	0.8766
Day 3	(0-level) vs. (1-level)	-0.0645	-0.402	0.9987
	(0-level) vs. (2-level)	0.0548	0.331	0.9995
	(0-level) vs. (4-level)	-0.2364	-1.530	0.6446
	(0-level) vs. (6-level)	-0.0263	-0.162	1.0000
	(0-level) vs. (8-level)	0.0270	0.164	1.0000
	(1-level) vs. (2-level)	0.1193	0.732	0.9780
	(1-level) vs. (4-level)	-0.1719	-1.132	0.8680
	(1-level) vs. (6-level)	0.0382	0.239	0.9999
	(1-level) vs. (8-level)	0.0916	0.566	0.9932
	(2-level) vs. (4-level)	-0.2912	-1.856	0.4295
	(2-level) vs. (6-level)	-0.0811	-0.493	0.9964
	(2-level) vs. (8-level)	-0.0278	-0.167	1.0000
	(4-level) vs. (6-level)	0.2101	1.370	0.7451
	(4-level) vs. (8-level)	0.2634	1.692	0.5368
	(6-level) vs. (8-level)	0.0533	0.327	0.9995
Day 5	(0-level) vs. (1-level)	0.0991	0.861	0.9556
	(0-level) vs. (2-level)	0.1562	1.338	0.7639

	(0-level) vs. (4-level)	0.1131	0.979	0.9246
	(0-level) vs. (6-level)	0.4942	3.836	0.0017
	(0-level) vs. (8-level)	0.5046	3.904	0.0013
	(1-level) vs. (2-level)	0.0572	0.478	0.9969
	(1-level) vs. (4-level)	0.0140	0.118	1.0000
	(1-level) vs. (6-level)	0.3951	3.008	0.0315
	(1-level) vs. (8-level)	0.4055	3.077	0.0255
	(2-level) vs. (4-level)	-0.0432	-0.360	0.9992
	(2-level) vs. (6-level)	0.3379	2.543	0.1119
	(2-level) vs. (8-level)	0.3483	2.613	0.0941
	(4-level) vs. (6-level)	0.3811	2.893	0.0442
	(4-level) vs. (8-level)	0.3915	2.963	0.0361
	(6-level) vs. (8-level)	0.0104	0.072	1.0000
Day 7	(0-level) vs. (1-level)	0.0445	0.447	0.9978
	(0-level) vs. (2-level)	0.3221	3.004	0.0319
	(0-level) vs. (4-level)	0.3629	3.344	0.0107
	(0-level) vs. (6-level)	0.7478	6.098	<.0001
	(0-level) vs. (8-level)	0.8239	6.597	<.0001
	(1-level) vs. (2-level)	0.2776	2.565	0.1060
	(1-level) vs. (4-level)	0.3185	2.908	0.0424
	(1-level) vs. (6-level)	0.7033	5.694	<.0001
	(1-level) vs. (8-level)	0.7885	6.202	<.0001
	(2-level) vs. (4-level)	0.0408	0.350	0.9993
	(2-level) vs. (6-level)	0.4257	3.277	0.0134
	(2-level) vs. (8-level)	0.5108	3.831	0.0018
	(4-level) vs. (6-level)	0.3848	2.939	0.0387
	(4-level) vs. (8-level)	0.4700	3.498	0.0062
	(6-level) vs. (8-level)	0.0852	0.583	0.9922

Table S2.

The statistical results of the number of aggressive behavior in black rockfish among recording days within specific treatment group.

Treatment	Contrast	Estimate	z.ratio	p.value
0-level	day 1 vs. day 3	-0.0270	-0.164	0.9984
	day 1 vs. day 5	-0.7784	-5.506	<.0001
	day 1 vs. day 7	-1.0423	-7.657	<.0001
	day 3 vs. day 5	-0.7514	-5.364	<.0001
	day 3 vs. day 7	-1.0152	-7.533	<.0001
	day 5 vs. day 7	-0.2638	-2.502	0.0596
1-level	day 1 vs. day 3	-0.7985	-3.979	0.0004
	day 1 vs. day 5	-1.3863	-7.440	<.0001
	day 1 vs. day 7	-1.7047	-9.409	<.0001
	day 3 vs. day 5	-0.5878	-4.215	0.0001
	day 3 vs. day 7	-0.9062	-6.841	<.0001
	day 5 vs. day 7	-0.3185	-2.908	0.0191
2-level	day 1 vs. day 3	-0.2924	-1.611	0.3725
	day 1 vs. day 5	-0.9424	-5.820	<.0001
	day 1 vs. day 7	-1.0403	-6.510	<.0001
	day 3 vs. day 5	-0.6500	-4.439	0.0001
	day 3 vs. day 7	-0.7480	-5.192	<.0001
	day 5 vs. day 7	-0.0980	-0.828	0.8414
4-level	day 1 vs. day 3	-1.5581	-6.333	<.0001
	day 1 vs. day 5	-1.9601	-8.207	<.0001
	day 1 vs. day 7	-1.9741	-8.273	<.0001
	day 3 vs. day 5	-0.4020	-3.033	0.0130
	day 3 vs. day 7	-0.4159	-3.147	0.0090
	day 5 vs. day 7	-0.0140	-0.118	0.9994
6-level	day 1 vs. day 3	-0.3926	-2.187	0.1268
	day 1 vs. day 5	-0.6235	-3.628	0.0016
	day 1 vs. day 7	-0.6337	-3.694	0.0013
	day 3 vs. day 5	-0.2309	-1.513	0.4297
	day 3 vs. day 7	-0.2412	-1.584	0.3880
	day 5 vs. day 7	-0.0103	-0.072	0.9999
8-level	day 1 vs. day 3	-0.1316	-0.768	0.8687
	day 1 vs. day 5	-0.4055	-2.513	0.0580

	day 1 vs. day 7	-0.3409	-2.085	0.1579
	day 3 vs. day 5	-0.2739	-1.764	0.2910
	day 3 vs. day 7	-0.2094	-1.329	0.5443
	day 5 vs. day 7	0.0645	0.440	0.9716

Table S3.

The statistical results of the number of aggressive behavior in fat greenling among treatments within specific recording day.

Record day	Contrast	Estimate	z.ratio	p.value
Day 1	(0-level) vs. (1-level)	-1.23676	-3.266	0.0139
	(0-level) vs. (2-level)	-1.60944	-4.408	0.0002
	(0-level) vs. (4-level)	-1.17007	-3.066	0.0264
	(0-level) vs. (6-level)	-0.89382	-2.259	0.2111
	(0-level) vs. (8-level)	-1.63142	-4.476	0.0001
	(1-level) vs. (2-level)	-0.37268	-1.597	0.6008
	(1-level) vs. (4-level)	0.06669	0.258	0.9998
	(1-level) vs. (6-level)	0.34294	1.230	0.8221
	(1-level) vs. (8-level)	-0.39465	-1.698	0.5328
	(2-level) vs. (4-level)	0.43937	1.845	0.4366
	(2-level) vs. (6-level)	0.71562	2.751	0.0657
	(2-level) vs. (8-level)	-0.02198	-0.105	1.0000
	(4-level) vs. (6-level)	0.27625	0.977	0.9253
	(4-level) vs. (8-level)	-0.46135	-1.946	0.3741
	(6-level) vs. (8-level)	-0.73760	-2.845	0.0506
Day 3	(0-level) vs. (1-level)	0.14953	0.610	0.9903
	(0-level) vs. (2-level)	-0.78593	-3.908	0.0013
	(0-level) vs. (4-level)	-0.60614	-2.925	0.0402
	(0-level) vs. (6-level)	0.05716	0.239	0.9999
	(0-level) vs. (8-level)	-0.70694	-3.471	0.0069
	(1-level) vs. (2-level)	-0.93546	-4.414	0.0001
	(1-level) vs. (4-level)	-0.75567	-3.471	0.0069
	(1-level) vs. (6-level)	-0.09237	-0.372	0.9991
	(1-level) vs. (8-level)	-0.85647	-3.995	0.0009
	(2-level) vs. (4-level)	0.17979	1.078	0.8902
	(2-level) vs. (6-level)	0.84309	4.110	0.0006
	(2-level) vs. (8-level)	0.07899	0.487	0.9966
	(4-level) vs. (6-level)	0.66329	3.142	0.0208
	(4-level) vs. (8-level)	-0.10080	-0.593	0.9915
	(6-level) vs. (8-level)	-0.76410	-3.680	0.0032
Day 5	(0-level) vs. (1-level)	-0.08269	-0.454	0.9976
	(0-level) vs. (2-level)	-0.38221	-2.244	0.2176

	(0-level) vs. (4-level)	-0.23002	-1.308	0.7810
	(0-level) vs. (6-level)	0.09015	0.474	0.9970
	(0-level) vs. (8-level)	-0.12921	-0.718	0.9798
	(1-level) vs. (2-level)	-0.29952	-1.802	0.4646
	(1-level) vs. (4-level)	-0.14732	-0.857	0.9566
	(1-level) vs. (6-level)	0.17284	0.927	0.9396
	(1-level) vs. (8-level)	-0.04652	-0.264	0.9998
	(2-level) vs. (4-level)	0.15219	0.954	0.9323
	(2-level) vs. (6-level)	0.47236	2.699	0.0754
	(2-level) vs. (8-level)	0.25300	1.542	0.6369
	(4-level) vs. (6-level)	0.32017	1.774	0.4826
	(4-level) vs. (8-level)	0.10080	0.593	0.9915
	(6-level) vs. (8-level)	-0.21936	-1.189	0.8422
Day 7	(0-level) vs. (1-level)	-0.30855	-2.134	0.2695
	(0-level) vs. (2-level)	-0.36865	-2.582	0.1016
	(0-level) vs. (4-level)	-0.36028	-2.519	0.1184
	(0-level) vs. (6-level)	-0.01198	-0.077	1.0000
	(0-level) vs. (8-level)	0.42986	2.459	0.1366
	(1-level) vs. (2-level)	-0.06010	-0.459	0.9975
	(1-level) vs. (4-level)	-0.05174	-0.394	0.9988
	(1-level) vs. (6-level)	0.29657	2.059	0.3093
	(1-level) vs. (8-level)	0.73840	4.463	0.0001
	(2-level) vs. (4-level)	0.00837	0.065	1.0000
	(2-level) vs. (6-level)	0.35667	2.507	0.1219
	(2-level) vs. (8-level)	0.79851	4.873	<.0001
	(4-level) vs. (6-level)	0.34831	2.444	0.1412
	(4-level) vs. (8-level)	0.79014	4.816	<.0001
	(6-level) vs. (8-level)	0.44183	2.533	0.1145

Table S4.

The statistical results of the number of aggressive behavior in fat greenling among recording days within specific treatment group.

Treatment	Contrast	Estimate	z.ratio	p.value
0-level	day 1 vs. day 3	-1.3863	-3.720	0.0011
	day 1 vs. day 5	-1.8632	-5.201	<.0001
	day 1 vs. day 7	-2.2216	-6.330	<.0001
	day 3 vs. day 5	-0.4769	-2.248	0.1106
	day 3 vs. day 7	-0.8353	-4.186	0.0002
	day 5 vs. day 7	-0.3584	-2.094	0.1549
1-level	day 1 vs. day 3	0.0000	0.000	1.0000
	day 1 vs. day 5	-0.7091	-3.232	0.0067
	day 1 vs. day 7	-1.2934	-6.379	<.0001
	day 3 vs. day 5	-0.7091	-3.232	0.0067
	day 3 vs. day 7	-1.2934	-6.379	<.0001
	day 5 vs. day 7	-0.5843	-3.716	0.0012
2-level	day 1 vs. day 3	-0.5628	-3.013	0.0138
	day 1 vs. day 5	-0.6360	-3.450	0.0031
	day 1 vs. day 7	-0.9808	-5.611	<.0001
	day 3 vs. day 5	-0.0732	-0.468	0.9660
	day 3 vs. day 7	-0.4180	-2.885	0.0204
	day 5 vs. day 7	-0.3448	-2.432	0.0711
4-level	day 1 vs. day 3	-0.8224	-3.691	0.0013
	day 1 vs. day 5	-0.9232	-4.206	0.0002
	day 1 vs. day 7	-1.4118	-6.817	<.0001
	day 3 vs. day 5	-0.1008	-0.593	0.9341
	day 3 vs. day 7	-0.5895	-3.841	0.0007
	day 5 vs. day 7	-0.4887	-3.287	0.0056
6-level	day 1 vs. day 3	-0.4353	-1.591	0.3837
	day 1 vs. day 5	-0.8792	-3.467	0.0030
	day 1 vs. day 7	-1.3398	-5.594	<.0001
	day 3 vs. day 5	-0.4439	-2.020	0.1804
	day 3 vs. day 7	-0.9045	-4.450	0.0001
	day 5 vs. day 7	-0.4605	-2.625	0.0430
8-level	day 1 vs. day 3	-0.4608	-2.453	0.0675
	day 1 vs. day 5	-0.3610	-1.880	0.2368

	day 1 vs. day 7	-0.1603	-0.799	0.8548
	day 3 vs. day 5	0.1008	0.593	0.9341
	day 3 vs. day 7	0.3015	1.680	0.3345
	day 5 vs. day 7	0.2007	1.094	0.6935
